# Effects of Robot-Aided Rehabilitation on the Ankle Joint Properties and Balance Function in Stroke Survivors: A Randomized Controlled Trial

**DOI:** 10.3389/fneur.2021.719305

**Published:** 2021-10-13

**Authors:** Xiaoxue Zhai, Qiong Wu, Xin Li, Quan Xu, Yanlin Zhang, Senchao Fan, Li-Qun Zhang, Yu Pan

**Affiliations:** ^1^Department of Rehabilitation, Beijing Tsinghua Changgung Hospital, Beijing, China; ^2^School of Clinical Medicine, Tsinghua University, Beijing, China; ^3^Department of Physical Therapy and Rehabilitation Science, University of Maryland, Baltimore, MD, United States; ^4^Department of Orthopaedics, University of Maryland, Baltimore, MD, United States; ^5^Department of Bioengineering, University of Maryland, College Park, MD, United States

**Keywords:** stroke, ankle, spasticity, stiffness, balance, robot

## Abstract

**Background:** Stroke survivors with impaired control of the ankle due to stiff plantarflexors often experience abnormal posture control, which affects balance and locomotion. Forceful stretching may decrease ankle stiffness and improve balance. Recently, a robot-aided stretching device was developed to decrease ankle stiffness of patient post-stroke, however, their benefits compared to manual stretching exercises have not been done in a randomized controlled trial, and the correlations between the ankle joint biomechanical properties and balance are unclear.

**Objective:** To compare the effects of robot-aided to manual ankle stretching training in stroke survivors with the spastic ankle on the ankle joint properties and balance function post-stroke, and further explore the correlations between the ankle stiffness and balance.

**Methods:** Twenty inpatients post-stroke with ankle spasticity received 20 minutes of stretching training daily over two weeks. The experimental group used a robot-aided stretching device, and the control group received manual stretching. Outcome measures were evaluated before and after training. The primary outcome measure was ankle stiffness. The secondary outcome measures were passive dorsiflexion ranges of motion, dorsiflexor muscle strength, Modified Ashworth Scale (MAS), Fugl-Meyer Motor Assessment of Lower Extremity (FMA-LE), Berg Balance Scale (BBS), Modified Barthel Index (MBI), and the Pro-Kin balance test.

**Results:** After training, two groups showed significantly within-group improvements in dorsiflexor muscle strength, FMA-LE, BBS, MBI (*P* < 0.05). The between-group comparison showed no significant differences in all outcome measures (*P* > 0.0025). The experimental group significantly improved in the stiffness and passive range of motion of dorsiflexion, MAS. In the Pro-Kin test, the experimental group improved significantly with eyes closed and open (*P* < 0.05), but significant improvements were found in the control group only with eyes open (*P* < 0.05). Dorsiflexion stiffness was positively correlated with the Pro-Kin test results with eyes open and the MAS (*P* < 0.05).

**Conclusions:** The robot-aided and manual ankle stretching training provided similar significant improvements in the ankle properties and balance post-stroke. However, only the robot-aided stretching training improved spasticity and stiffness of dorsiflexion significantly. Ankle dorsiflexion stiffness was correlated with balance function.

**Clinical Trial Registration:** www.chictr.org.cn ChiCTR2000030108.

## Introduction

Stroke is a leading cause of mortality, and approximately 2.5 million people experience a stroke annually in China (1). Stroke survivors have abnormal balance function due to spasticity, muscle weakness, sensory loss, and/or motor dysfunction (2–4). Structural changes of muscle fibers and connective tissue in stroke patients may result in a reduction in joint range of motion (ROM) and a clinical contracture for lacking mobilization and serious spasticity (5–9). Previous studies have demonstrated that stroke patients with impaired ankle control due to stiff plantarflexors and weak dorsiflexors often have a high fall rate (4, 10) because the ankle is crucial to control the location of the body's base of support and assist in controlling balance (11). Maintaining balance relies on well-controlled contraction of dorsiflexors and plantarflexors and specific ankle ROM (4, 12). Therefore, alleviating ankle muscle stiffness, and improving the muscles' soft-tissue extensibility and viscoelastic properties are important rehabilitation goals for stroke survivors in reestablishing balance function (13).

Many treatments to improve balance ability in stroke survivors are aimed at improving the posture control of the trunk and lower limbs including the use of strengthening exercises, functional neuromuscular stimulation, and visual feedback balance training (14–16). Ankle stretching exercises are also widely utilized to prevent and treat limited ankle ROM post-stroke to improve balance ability. Previous studies have demonstrated that higher resistance torque, increased joint stiffness, and decreased ankle ROM characterized by stroke survivors improved after completing passive stretching exercises (17–21). The aims of stretching exercises are to increase soft-tissue extensibility, normalize muscle tone, improve function and reduce pain (17, 22, 23). Passive ankle stretching can be manually applied by physical therapists or by using a stretching board, or by robotic systems (20, 23–26). Some of the factors that have limited clinic therapeutic regimens including cost, labor-intensive manual provision, availability of physical therapists, and limited access to clinical facilities. In practice, there are differences among therapies in the actual effects of manual stretching training, such as subjective judgment about the severity of spasticity, intensity, frequency, and duration of the manual stretching exercises.

Recently, an intelligent robot-aided stretching device was developed to decrease ankle stiffness of patients with neurological impairment due to stroke, spinal cord injury, multiple sclerosis, or cerebral palsy. Significant improvements were found in the ROM, maximum voluntary contraction, ankle stiffness, and comfortable walking speed (20, 27–29). The stretching velocity of this device decreases as resistance increasing, and will hold the ankle joint at the extreme position for a while to let stress relaxation occur when the predefined resistance torque is reached. By using this control strategy, the stretching device moves quickly in the middle (non-spastic) ROM and slows down in the stiffer part of the ROM, while never exceeding predefined stretching torques (19). Robotic adaptive stretching may be a quantitative stretching alternative therapy (30).

At present, there is a lack of RCT comparing the effects of robot-aided to manual ankle stretching training on the ankle joint properties and balance function post-stroke. The primary aim of this study was to evaluate the effects of the intelligent robot-aided stretching and manual stretching therapies on the ankle properties and balance function post-stroke. A secondary aim was to study how ankle stretching affects balance. We hypothesized ankle stretching would improve balance function for changing the neural and musculoskeletal characteristics of the ankle joint, and there may be different mechanisms between manual and intelligent stretching. A third aim was to investigate the relationship between ankle stiffness and balance function post-stroke.

## Methods

### Trial Design

This is an assessor-blinded, randomized controlled trial. The aim was to compare the effects of robot-aided to manual ankle stretching training in stroke survivors with the spastic ankle on the ankle properties and balance function post-stroke, and further explore the correlations between the stiffness of the ankle and balance. The study was conducted according to the tenets of the Declaration of Helsinki, the guidelines for Good Clinical Practice, and the Consolidated Standards of Reporting Trials (CONSORT), approved by the local Ethics Committee “Beijing Tsinghua Chang Gung Hospital Medical Ethics” (18172-0-01), and registered at clinical trial (ChiCTR2000030108).

### Participants

This RCT was conducted at the Beijing Tsinghua Chang Gung Hospital in China. Inpatients with stroke in the rehabilitation department of the hospital were recruited between May 2019 and November 2019. The inclusion criteria were: (1) ages between 18 and 75 years; (2) first-ever stroke with less than 6 months duration of spasticity of the affected ankle (Modified Ashworth Scale, MAS: 1-3 points); (3) medically stable; and (4) ability to stand independently without aids for at least 1 minute. Exclusion criteria were: communication problems, dementia based on clinical diagnosis, comorbidities affecting motor performance such as orthopedic, arthritic, inflammatory conditions that could influence balance, and limited ankle movement.

### Interventions

Subjects in the experimental and control group had 10-session stretching training using the intelligent robot-aided stretching or manual stretching respectively (five times a week over 2 weeks, 20 minutes/session). During the 2-week period, both groups continued movement exercises for ankle mobility and strength.

### Experimental Setup

**Figure 1:**
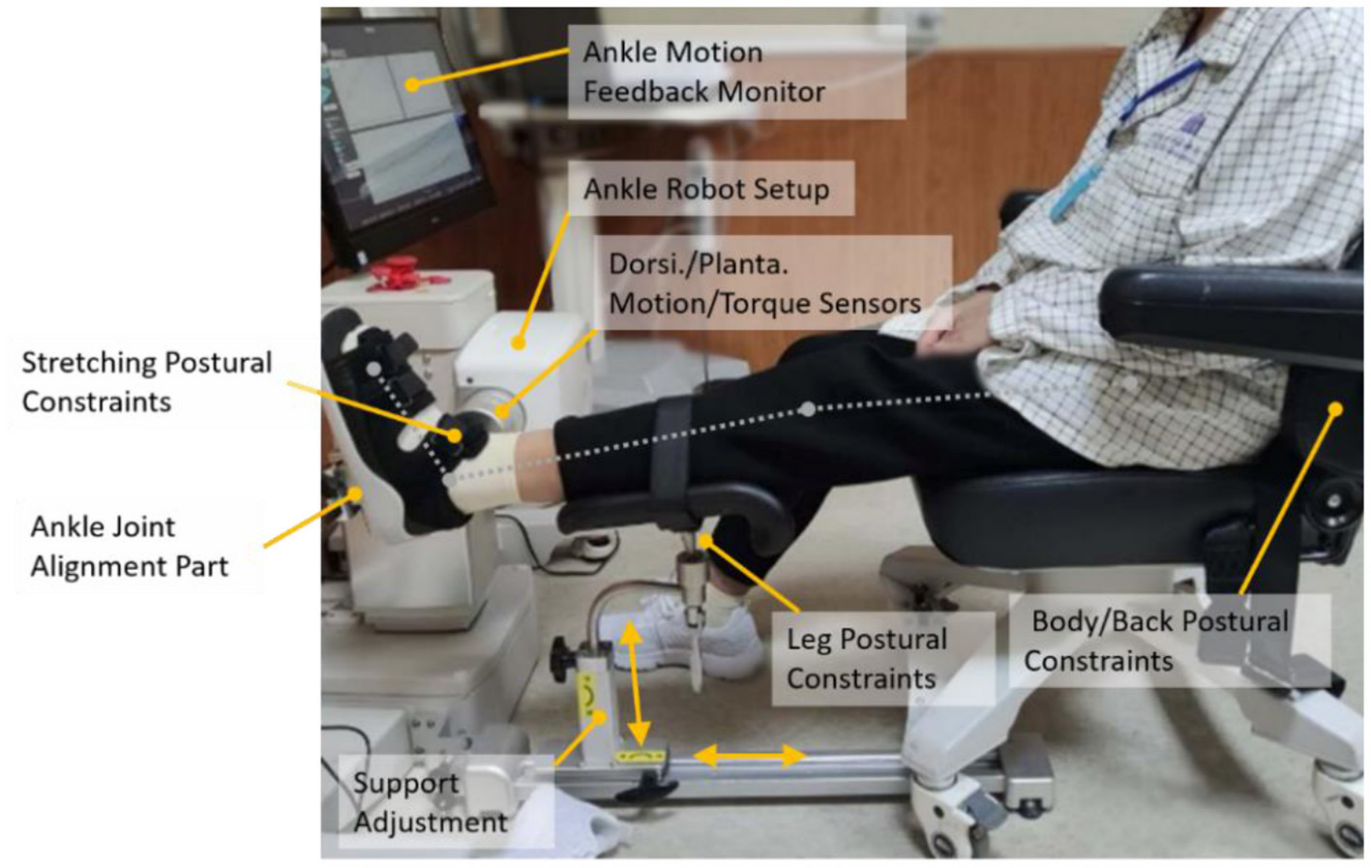
A subject seated in the ankle rehabilitation robot device.

An ankle rehabilitation robot (Beijing LTK Science and Technology Co., Ltd., Beijing, China) was used for intervention and outcome evaluations. While the subject was comfortably seated, the leg of the subject was strapped to leg support with the knee at 30° flexion and the foot was strapped onto a footplate with ankle dorsiflexion (DF) at 0°. The foot was secured to a footplate at the dorsal side and the heel using adjustable straps. The footplate was fixed to the motor shaft, and a torque sensor was aligned with the motor shaft to measure the ankle joint torque (Figure 1). The ankle stretching device was clamped to the chair to avoid movement of the device during stretching (18).

### Stretching Protocol

The ankle rehabilitation robot was driven by a servomotor controlled by a digital signal processor (20). Briefly, the stretching velocity was inversely proportional to the joint resistance torque, with the control adjusted at 2,000 Hz. The maximum stretching velocity was set at 12°/s (31). Typical stretching parameters were 15 to 20 Nm peak resistance torque in dorsiflexion, 5 to 10 Nm peak resistance torque in plantarflexion, and a 5-second holding period at the extreme positions. An experienced physiotherapist adjusted the peak resistance torque for each session based on manual stretching and feedback from the subject during the stretching therapy. When receiving the intelligent stretching exercises, the subjects were required to look at the display screen where an “ankle joint” moves from dorsiflexion to plantarflexion as the real dynamic stretching simultaneously and try to feel the ankle movement. The control group received stretching sessions in a clinic by the appointed physiotherapist. The positive range of motion (PROM) of the ankle was measured using a goniometer to ensure the safety of manual stretching before manual stretching exercises. Subjects remained as relaxed as possible while the physiotherapist stretching the paretic ankle from plantar to dorsiflexion in the sagittal plane slowly, and a 5-second holding period at the extreme dorsiflexion positions. There is no break in the process of manual or intelligent stretching training.

### Outcomes

Clinical and demographic data were collected at enrollment. Subjects were evaluated before and after the interventions by a designated physiotherapist blinded to the group assignment. The primary outcome of the study was the change of stiffness of the ankle after the training, due to its relevance in physiologic control of the ankle. The secondary outcomes of the study were divided into three categories: biomechanical evaluations, clinical evaluations, and the Pro-Kin balance test (Pro-Kin254P, TecnoBody Company, Italy). The assessments included pre-assessment (baseline, before the first exercise session), post-assessment (after the tenth exercise session). The assessment sessions were done at the same time of the day with the assessments in the same order.

### Biomechanical Evaluations

Evaluations included the DF PROM (passive ranges of motion measured in dorsiflexion direction movement), DF and PF stiffness (stiffness measured in dorsiflexion and plantarflexion direction movement), and dorsiflexor muscle strength. ROM and muscle strength were measured using the HogganMicroFET3 portable device (Hoggan Health Industries, Inc. Salt Lake City, USA). The Stiffness is defined as the ratio of ground reaction moment to angular deflection of the specific joint. Ankle stiffness measured in DF or PF passive movement was assessed as K = ΔT/Δθ, where K (Nm/°) was the quasi-static stiffness and ΔT was the passive torque increment during a certain amount of ankle angular movement (Δθ). As Δθ becomes infinitely small, the quasi-static stiffness approaches the slope of a tangential line of the torque-angle curve at a specific ankle position (32, 33). The peak stretching velocity in this study was set at 5°/s to avoid inducing reflex responses (21). The biomechanical evaluations using the ankle robot have been used and validated in several previous studies (34, 35). Quasi-static stiffness of the ankle plantarflexor (DF stiffness) was evaluated at 10° of DF and that of the ankle dorsiflexor (stiffness measured in PF direction movement, PF stiffness) at 30° of PF for the PROM of the subjects in the two groups all meet this criterion.

### Clinical Evaluations

Each subject completed the following functional assessments during clinical evaluation sessions. MAS (0–5 points, with higher scores indicating worse spasticity) was used to measure the calf muscle hypertonia (36). Fugl-Meyer Motor Assessment of Lower Extremity (FMA-LE) (0–34 points) was used to evaluate the sensorimotor function of the lower limbs (37). The Berg Balance Scale (BBS) (0–56 points) was used to evaluate the balance function (38). The Modified Barthel Index (MBI) (0–100 points) was used to measure the activities of daily living (ADL) (39).

### Balancing Test

**Figure 2:**
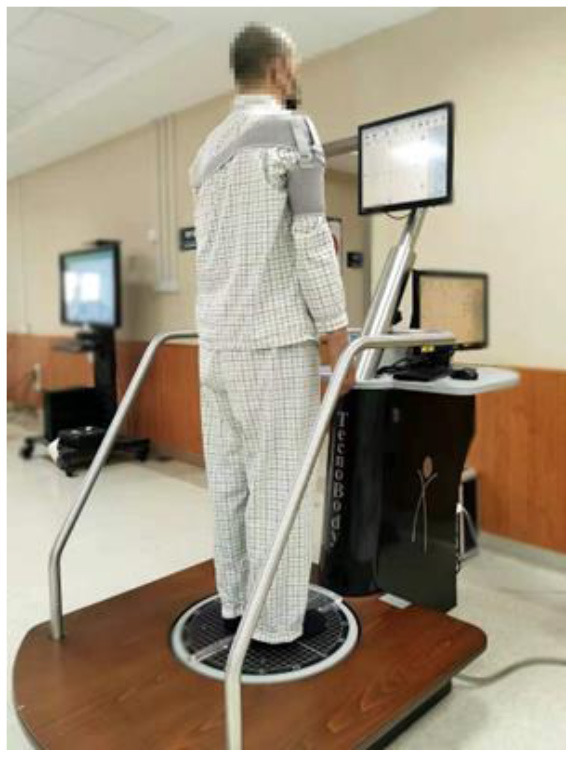
Static Balance Assessed by the Pro-Kin System.

This study also used a Balancing Instrument (Pro-Kin254P, TecnoBody Company, Italy) to assess balance function, based on the instantaneous data of postural sway using the force platform from movements of the center of pressure (COP) (16) (Figure 2). The COP is a weighted average of all the pressures over the surface area in contact with the ground. This is a valid and reliable device that measures static and dynamic balance function (40, 41). The force platform consists of multiple strain gauges placed under a circular surface of 50 cm of diameter at 120° to each other and has a 20 Hz sampling frequency (42). When subjects were standing on the platform, the COP sway was documented. The COP measures demonstrate where a subject's pressure is located in both the x- and y-axes. An increase in COP in either the F/B or M/L direction is indicative of postural disturbance. Subjects were required to stand statically on the force platform, and maintain visual focus on an “X mark” placed on an eye-level screen from their face. The position of the feet on the platform was standardized using a V-shaped frame. Each subject performed two standing tests lasting 30 seconds each: One test with eyes open (EO) and one with eyes closed (EC). There were six outcome variables: trajectory lengths (measured in mm), elliptical trajectory (measured in mm^2^), standard deviation medial/lateral (M/L SD measured in mm), standard deviation forward/backward (F/B SD measured in mm), average speed medial/lateral (M/L AS measured in m/s), and average speed forward/backward (F/B AS measured in m/s). Smaller values of the six parameters indicated the subject had a better balance function (43).

### Sample Size

The sample size calculation was conducted using G^*^Power 3.1.7 (http://www.gpower.hhu.de/). The effect size was estimated using our pilot data regarding decreases in DF stiffness after training (experimental group vs control group: 0.61 ± 0.21 vs. 0.31 ± 0.27) would be able to reveal a large effect size of Cohen's d = 1.24, at a power of 0.8 and an α level of 0.05 assuming a non-directional hypothesis. Thus, in the current study, a large effect size f = 0.4 was assumed in the Mann–Whitney U test model, with an α value of 0.05, power of 0.8, and an attrition rate of 10%, the minimum required sample size was estimated to be 18 subjects for this study.

### Randomization and Blinding

After recruited subjects presented written informed consent, they were randomly assigned into the experimental group or control group in 1:1 ratio by drawing lots. The lots were designated as “experimental” or “control” by stratified randomization with random numbers generated from statistical software and presented in sealed opaque envelopes. Each subject received a sealed envelope that indicated the group they were assigned to. The researchers in charge of recruitment and randomization procedures were different, and the designated therapist was responsible for the assessment was kept blinded to the group allocation throughout the trial.

### Statistical Analysis

Baseline characteristics were compared between the two groups by using Fisher's exact test for categorical variables and the Mann-Whitney U test for continuous and ordinal variables. The continuous variables were tested using the Shapiro-Wilk test to verify whether they met the normal distribution and using the homogeneity of variance test. Change with each intervention and during an observation period of two weeks were examined between the groups with a Mann-Whitney U test. Bonferroni corrections were applied to account for multiple comparisons (α = 0.0025) to reduce the probability of Type-I error. The Wilcoxon Signed Rank test was used to compare pre-and post-intervention measurements in each group. Furthermore, to more deeply understand the effects of ankle stiffness on balance function, the Spearman correlation analysis was performed for testing the association between stiffness and the Pro-Kin test, and the Kendall rank correlation coefficient (τ) for the correlation between MAS and ankle stiffness (effects were considered significant if *P* < 0.05). Under a small sample size, T-distribution was used to compute a 95% confidence interval (95% CI). All statistical analyses were performed with SPSS version 21.0. (IBM Corporation, Armonk, NY, USA).

## Results

### The Flow of the Trial and Baseline Characteristics of Subjects

**Figure 3:**
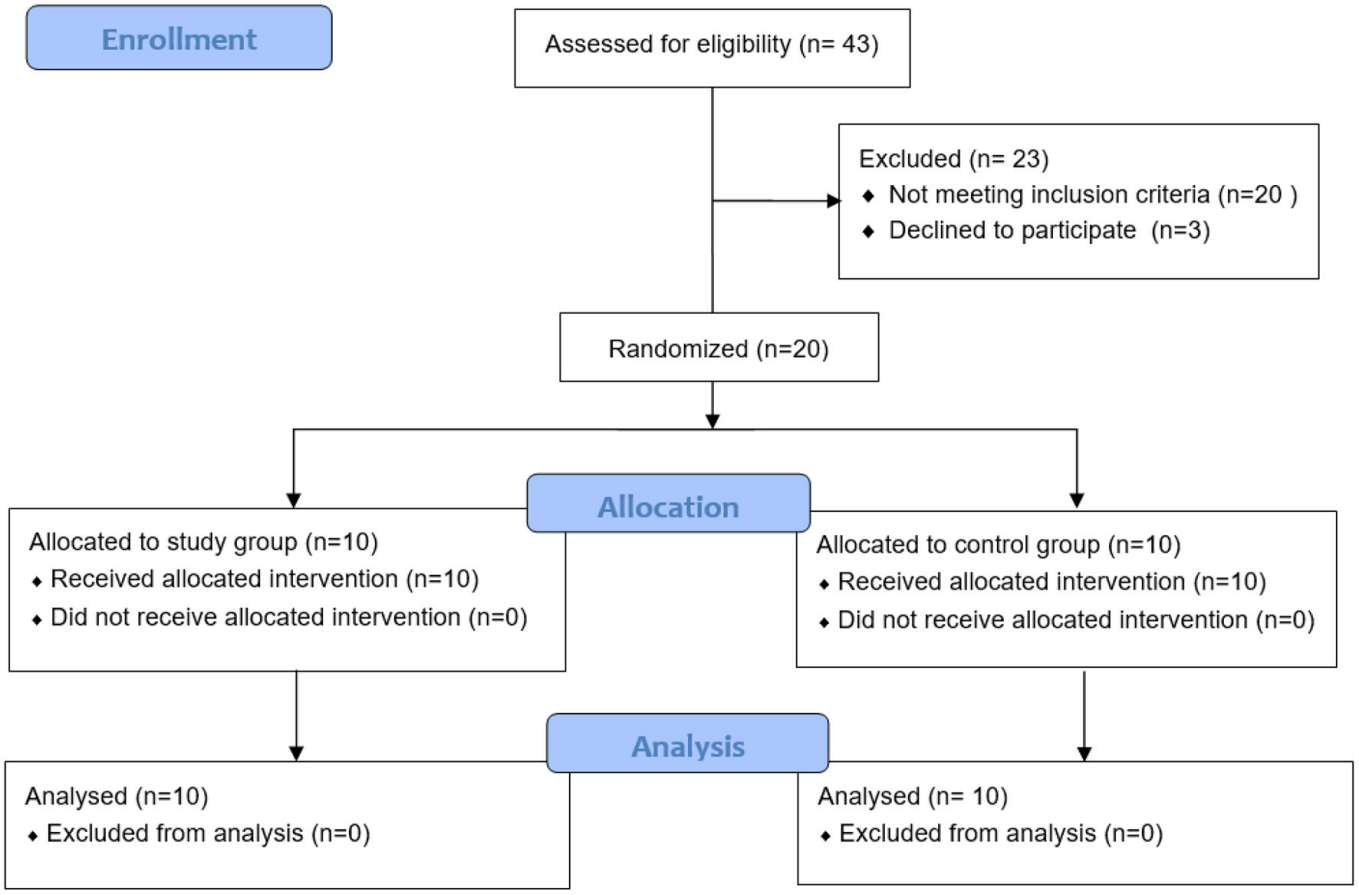
CONSORT patient flow throughout the study.

From May 2019 to November 2019, all inpatients in the rehabilitation department were screened. Of these, 43 stroke patients with ankle spasticity were eligible for evaluation. Among these subjects, 20 subjects did not meet the inclusion criteria, and 3 subjects declined to participate in this study (see Figure 3 for more details). A total of 20 subjects were recruited to the study, including 10 subjects randomized to the experimental group, and 10 subjects randomized to the control group. All enrolled subjects completed the 2-week training, and there were no dropouts or adverse events. There were no significant differences in subjects' characteristics between the two groups (Table 1).

**Table 1:** Baseline Characteristics of the Subjects^a^.

**Parameters**	**Experimental group**	**Control group**	***P***
	**(***n*** = 10)**	**(***n*** = 10)**	
Age (year)	61.90 ± 9.62	60.00 ± 6.62	0.288^c^
Duration post-stroke (day)	54.20 ± 33.85	58.10 ± 50.20	0.650^c^
Sex (M/F)	9/1	9/1	1.00^b^
Cerebral infarction/cerebral hemorrhage (case)	10/0	10/0	1.00^b^
Side of lesion (right/left, case)	7/3	7/3	1.00^b^
Height (*m*)	1.68 ± 0.05	1.69 ± 0.04	0.568^c^
Weight (*kg*)	74.70 ± 9.71	70.40 ± 5.40	0.120^c^
BMI (*kg*/*m*^2^)	26.33 ± 3.65	24.45 ± 1.72	0.082^c^

*BMI, Body Mass Index; M, male; F, female*.

a *Values are mean ± standard deviation, or number*.

b *Fisher's exact test*.

c *Mann–Whitney U tests between groups for baseline values. There were no significant differences between groups at baseline for clinical characteristics*.

### Biomechanical Evaluations: DF PROM, DF Muscle Strength and Joint Stiffness

**Figure 4:**
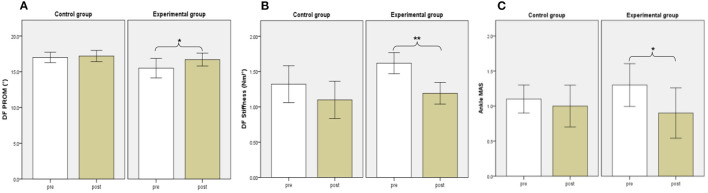
Biomechanical and Clinical evaluations between groups. DF PROM **(A)**, DF Stiffness **(B)**, Ankle MAS **(C)**. White and brown bars indicate pre-, and post-assessment. Error bars indicate standard error of the corresponding average (^*^p < 0.05 and ^**^P < 0.01). DF, dorsiflexion; PROM, positive range of motion; MAS, Modified Ashworth Scale.

Before training, there were no significant differences in DF PROM, DF muscle strength, or DF and PF stiffness between the two groups. The DF muscle strength increased significantly after the 2-week training period in the control group and experimental group (*P* = 0.005, and 0.005, respectively). Besides, significant decreases in DF stiffness and improvements in DF PROM were found for subjects in the experimental group (*P* = 0.008, and 0.041, respectively), but not in the control group (*P* = 0.139, and 0.157, respectively). No significant differences in biomechanical evaluations were found between the two groups after training (*P* > 0.0025) (Table 2) (Figure 4).

**Table 2:** Biomechanical Properties at pre- and post-training between two groups.

	**Experimental group**			**Control group**			**Between-Group** **Difference in Change**
**Variable**	**Pre**	**Post^a^**	**Change**	**Pre**	**Post^a^**	**Change**	***P*** ^b^
	**Mean ± SD**	**Mean ± SD**	**Mean (LB; UB) 95%CI**	**Mean ± SD**	**Mean ± SD**	**Mean (LB; UB) 95%CI**	
DF PROM (°)	15.50 ± 2.17	16.70 ± 1.42^*^	1.20 (−0.14;2.54)	17.00 ± 1.15	17.20 ± 1.23	0.20 (−0.10;0.50)	0.108
DF Strength (N)	102.50 ± 44.54	132.82 ± 43.44^**^	30.32 (10.73;49.91)	101.4 ± 59.71	123.80 ± 58.55^**^	22.46 (6.55;38.37)	0.596
DF Stiffness (Nm/deg)	1.62 ± 0.24	1.19 ± 0.24^**^	−0.43 (−0.61;−0.24)	1.32 ± 0.41	1.10 ± 0.42	−0.22 (−0.53;0.08)	0.472
PF Stiffness (Nm/deg)	0.21 ± 0.04	0.19 ± 0.03	−0.02 (−0.05;0.02)	0.21 ± 0.07	0.24 ± 0.09	0.03 (−0.05;0.10)	0.622

*SD, standard deviation; CI, confidence interval. DF or PF stiffness is stiffness measured in dorsiflexion or plantarflexion passive movement*.

a *Comparison between pre- and post-training values in each group with Wilcoxon signed rank test*:

* *P < 0.05*,

** *P < 0.01*.

b P values indicate significance level of between-group differences in change with Mann-Whitney U test: according to the Bonferroni correction, ^***^P < 0.0025.

### Clinical Evaluations

There were no significant differences in MAS, FMA-LE, BBS, or MBI before training between the two groups. The FMA-LE, BBS, and MBI increased significantly after the 2-week training period in the control group (*P* = 0.005, 0.007, and 0.041, respectively). We also found significant improvement in FMA-LE, BBS, and MBI in the experimental group (*P* = 0.007, 0.012, and 0.007, respectively). Besides, significant decreases were found in MAS for subjects in the experimental group (*P* = 0.046) but not in the control group (*P* = 0.317). No significant differences in clinical evaluations were found between the two groups after training (*P* > 0.0025) (Table 3) (Figure 4).

**Table 3:** Clinical evaluations at pre- and post-training between two groups.

	**Experimental group**			**Control group**			**Between-Group** **Difference in Change**
**Variable**	**Pre**	**Post^a^**	**Change**	**Pre**	**Post^a^**	**Change**	***P*** ^b^
	**Mean ± SD**	**Mean ± SD**	**Mean (LB; UB) 95%CI**	**Mean ± SD**	**Mean ± SD**	**Mean (LB; UB) 95%CI**	
MAS	1.30 ± 0.48	0.90 ± 0.57^*^	−0.4 (−0.77;−0.03)	1.10 ± 0.32	1.00 ± 0.47	−0.10 (−0.33;0.13)	0.131
FM-LE	26.50 ± 5.36	29.30 ± 4.42^**^	2.8 (1.50;4.10)	24.80 ± 7.25	27.60 ± 6.31^**^	2.80 (1.02;4.58)	0.587
BBS	41.40 ± 13.74	45.00 ± 13.87^*^	3.60 (0.28;6.93)	43.40 ± 10.23	46.70 ± 9.93^**^	3.30 (1.33;5.27)	0.644
MBI	59.50 ± 15.89	75.00 ± 14.14^**^	15.50 (8.06;22.93)	75.50 ± 19.21	80.00 ± 17.95^*^	4.50 (0.56;8.44)	0.015

*SD, standard deviation; CI, confidence interval; MAS, Modified Ashworth Scale; FMA-LE, Fugl-Meyer Motor Assessment of Lower Extremity; BBS, Berg Balance Scale; PASS, Postural Assessment Scale for Stroke Patients; 6 MWT, 6-minute walk test; MBI, Modified Barthel*.

a *Comparison between pre- and post-training values in each group with Wilcoxon signed rank test*:

* *P < 0.05*,

** *P < 0.01*.

b P values indicate significance level of between-group differences in change with Mann-Whitney U test: according to the Bonferroni correction, ^***^P < 0.0025.

### Balancing Test Results

There was no significant difference between the two groups in the Pro-Kin balance test before training. The ellipse area, trajectory length, M/L SD, and F/B AS with closed eyes and F/B SD with opened eyes decreased significantly after the 2-week training period in the experimental group (*P* = 0.005, 0.013, 0.012, 0.005, and 0.041, respectively). The trajectory length and M/L AS with opened eyes decreased significantly after the 2-week training period in the control group (*P* = 0.022, and 0.042, respectively). No significant difference in the Pro-Kin balance test results was found between the two groups after training (*P* > 0.0025) (Table 4).

**Table 4:** Pro-Kin balance test results at pre- and post-training between two groups.

	**Experimental group**			**Control group**			**Between-Group** **Difference in Change**		
**Variable**	**Pre**	**Post^a^**	**Change**	**Pre**	**Post^a^**	**Change**	**P^b^**
	**Mean ± SD**	**Mean ± SD**	**Mean (LB; UB) 95%CI**	**Mean ± SD**	**Mean ± SD**	**Mean (LB; UB) 95%CI**	
Eyes Closed							
Ellipse Area *mm*^2^	1, 396.10 ± 1, 085.48	847.70 ± 486.15^**^	−548.40(−1024.58;−72.22)	2, 431.50 ± 2, 569.09	1, 371.00 ± 1, 236.41	−1, 059.50(−2, 748.31;629.31)	1.000
Trajectory Length *mm*	962.40 ± 344.94	820.80 ± 280.43^*^	−141.60(−254.13;−29.07)	1, 141.30 ± 6, 13.22	918.00 ± 522.08	−223.30(−705.04;258.44)	0.821
F/B SD	7.40 ± 1.90	6.80 ± 2.53	−0.60(−2.19;0.99)	10.20 ± 4.76	7.60 ± 2.72	−2.60(−5.87;0.67)	0.340
L/M SD	10.10 ±5.34	6.10 ± 2.77^*^	−4.00(−7.05;−0.95)	10.50 ± 6.00	9.10 ± 4.79	−1.40(−6.04;3.24)	0.068
F/B AS mm/sec	22.30 ± 8.37	18.60 ± 7.11^**^	−3.70(−5.49;−1.91)	23.50 ± 14.87	19.30 ± 14.12	−4.20(−13.77;5.37)	0.381
L/M AS mm/sec	18.90 ± 7.94	17.50 ± 7.86	−1.40(−6.21;3.41)	19.50 ± 13.30	15.40 ± 11.11	−4.10(−15.70;7.50)	0.909
Eyes Open							
Ellipse Area *mm*^2^	755.50 ± 659.29	518.90 ± 224.25	−236.60(−654.05;180.85)	713.20 ± 450.40	533.40 ± 201.92	−179.80(−478.82;119.22)	0.734
Trajectory Length *mm*	585.30 ± 188.54	458.70 ± 122.65	−126.60(−277.81;24.61)	539.30 ± 182.93	459.40 ± 126.06^*^	−79.90(−169.86;10.06)	0.705
F/B SD	5.80 ± 2.15	4.50 ± 0.85^*^	−1.30(−2.47;−0.13)	5.80 ± 1.75	5.10 ± 0.88	−0.70(−1.92;0.52)	0.301
L/M SD	7.10 ± 4.38	6.10 ± 1.73	−1.00(−3.92;1.92)	6.10 ± 2.08	5.90 ± 2.13	−0.20(−1.78;1.38)	0.817
F/B AS mm/sec	13.60 ± 5.46	10.80 ± 2.62	−2.8(−7.59;1.99)	11.30 ± 5.06	10.20 ± 3.97	−1.10(−3.25;1.05)	0.939
*L*/*M AS mm*/*sec*	11.00 ± 2.91	9.10 ± 4.31	−1.90(−5.07;1.27)	11.10 ± 4.12	9.00 ± 2.00^*^	−2.10(−4.40;0.20)	0.539

*SD, standard deviation; CI, confidence interval; AS, Average speed; F/B, Forward/Backward; M/L, Medial/Lateral direction*.

a *Comparison between pre- and post-training values in each group with Wilcoxon signed rank test*:

* *P < 0.05*,

** *P < 0.01*.

b P values indicate significance level of between-group differences in change with Mann-Whitney U test: according to the Bonferroni correction, ***P < 0.0025.

### Correlations Between the Stiffness of the Ankle and the Balance Function

**Figure 5:**
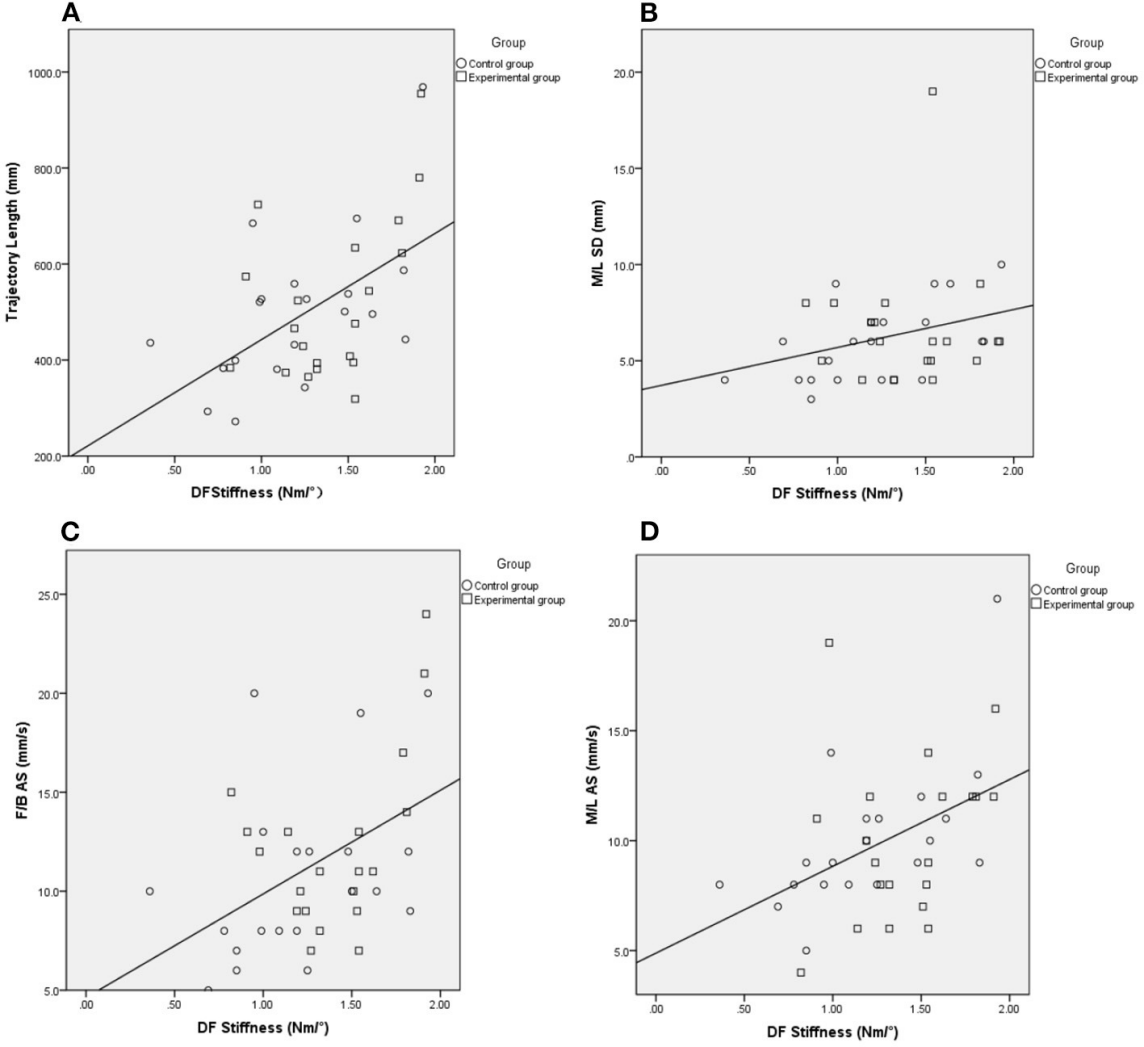
The Spearman correlation analysis between DF stiffness and outcomes of the Pro-Kin test with eyes open. Trajectory length **(A)**, M/L SD **(B)**, F/B AS **(C)**, M/L AS **(D)**, Circle and Square indicate control, and experimental group. EO, Eye Open; F/B, Forward/Backward; M/L, Medial/Lateral; AS, Average Speed; SD, Standard Deviation.

Regarding the two groups as a whole, we further explore the correlations between ankle stiffness and balance. The DF stiffness was significantly correlated with the results of the Pro-kin balance test with opened eyes, including the trajectory length, M/L SD, F/B AS, M/L AS (γ = 0.464, *P* = 0.003; γ = 0.313, P = 0.049; γ = 0.386, P=0.014; γ = 0.466, *P* = 0.002, respectively). The DF stiffness was also significantly correlated with MAS (τ = 0.265, *P* = 0.041) (Table 5) (Figure 5).

**Table 5:** Correlations Between stiffness and Pro-Kin balance test results when open eyes.

	**DF stiffness**	**P**	**PF stiffness**	**P**
MAS	0.265	0.041^*^	−0.133	0.317
Ellipse Area	0.262	0.102	−0.285	0.074
Trajectory Length	0.464	0.464	−0.308	0.053
F/B SD	0.155	0.339	−0.228	0.157
M/L SD	0.313	0.049^*^	−0.277	0.084
F/B AS	0.386	0.014^*^	−0.206	0.202
M/L AS	0.466	0.002^**^	−0.264	0.100

*Spearman's coefficient (γ) was used to estimate the correlation between stiffness (K) and the Pro-Kin system results, and the Kendall rank correlation coefficient (τ) was used to estimate the correlation between stiffness (K) and MAS. Values are γ or τ*.

* *P < 0.05*,

** *P < 0.01*.

## Discussion

The RCT showed significant within-group improvements in DF muscle strength, motor function of lower limbs, balance function, and activities of daily living after a 10-session training in two groups. However, the experimental group showed additional improvements in the DF stiffness, DF PROM, and MAS. Between-group comparisons represented no differences in all outcome measures. We believe the intelligent stretching robot could be an effective and safe alternative to manual stretching for therapists. Also, the intelligent stretching robot has the potential to use in stretching the ankle with spasticity and/or contracture regularly without the daily involvement of clinicians or physiotherapists.

Several studies have already been shown that continuous passive stretching can effectively reduce ankle stiffness (18, 20, 44). Another study further demonstrated that the repeated passive stretching can decrease spasticity through a combination of reflexive and mechanical factors for stroke survivors (45). Previous studies have shown that the change in mechanical properties of tendons depends on the stretching protocol (46). Stretching exercise under intelligent control has been effectively used to decrease ankle contracture and/or spasticity in stroke survivors (18, 20, 47). The intelligent stretching device was driven by a servomotor controlled by a digital signal processor (20). Briefly, the stretching velocity was inversely proportional to the joint resistance torque. The torque limits of plantar and dorsiflexion were preset before the stretching exercises. Once reaching the predefined resistance torque peak, the joint will be held at the extreme position for stress relaxation in a preset period (18, 32). This method may overcome potential viscoelastic responses and alter the muscle-tendon properties (48). Since the outcome of manual stretching might depend on the ability of the therapist to measure the limits of the ROM or “end feel” (19), which could not provide lasting and effective stretching. In addition, high-intensity stretching can induce a physiological response within the muscle-tendon unit and enhance neuroplasticity (49–52). Thus, the intelligent stretching robot can offer ideal exercise intensity, frequency, and duration (53, 54), while it is not feasible for the limited availability of physical therapists to deliver laborious manual therapy. This study demonstrated that improvements after the intelligent stretching of the spastic ankles post-stroke were consistent with previous research, including increased ROM and muscle strength, decreased ankle stiffness and spasticity (19, 55).

In this study, the Pro-Kin was used to quantitatively evaluate the static balance of subjects, excluding the influences of the hip and stride strategy, and further explore the role of ankle strategy in posture control independently (56). The experimental group had significant improvements in balance tests with eyes open and closed, while the control group only improved with eyes open, which suggested that two kinds of stretching models might take different mechanisms to improve balance function.

Balance disorders of stroke survivors have various causes, such as muscle weakness, limited range of motion, spasticity, sensory changes, loss of coordination, and impaired central integration. In particular, stiff plantar flexors and weak dorsiflexors on the affected side increasing the risk to cause the muscular imbalance of the ankle (57). In our study, M/L SD in the experimental group and M/L AS in the control group decreased significantly. We assumed that two kinds of stretching exercises could change the ankle properties, and can improve balance in the M/L direction, for improving the symmetry of weight-loading on the lower limbs by increasing the contribution of the paretic limb in stroke survivors. The intelligent stretching could forcefully, safely, and repeatedly stretch the ankle to its extreme positions in the sagittal plane resulting in structural changes in the viscoelastic properties of the muscles and connective tissues. This method can further reduce ankle stiffness and increasing ROM and improving the stability of the ankle joint (19–21), therefore the movement of COP decreased significantly in sagittal planes with eyes closed and opened in our experimental group. Adequate balance relies on an accurate perception of physical input from the visual, proprioceptive, and vestibular systems (58). Stroke survivors exhibited obvious decreased postural stability, especially without visual feedback. Proprioception around the ankle joint resulting from sensory inputs (e.g., from cutaneous receptors, muscle-spindle receptors, and Golgi tendon organ located in muscles, tendons, and ligaments) is damaged post-stroke, which impairs the ankle strategy (59). In this study, the M/L SD and F/B AS decreased significantly in the experimental group with eyes closed. We hypothesized the robot-aided cyclic stretching could enhance proprioception of ankle joints effectively, and the balance of patients with eyes closed is also improved even in the visual feedback removal (60). While the control group did not have significant improvements in balance function with eyes closed. Compared with manual stretching exercises, the intelligent stretching device can provide feedback for patients. The strength of stretching force and range of motion of the ankle were displayed in the interface accompanied by a real-time ankle animation. This visual feedback might provoke the recovery of the damaged central nervous system (CNS) (61). Besides, this system contains different user-friendly modes, such as the evaluation of biomechanical properties, and the training mode. Those user-friendly features might attract patients to participate in the training and accelerate the recovery of balance function.

An ankle strategy is typically used on a solid base supporting a small amount of body sway (56, 62). This study further explored the correlation between ankle stiffness and the Pro-Kin balance test results with opened eyes. The findings showed that the stiffness of dorsiflexion was positively related to trajectory length, M/L SD, F/B AS, and M/L AS when opened eyes. However, those tests showed no significant correlation between PF stiffness and the Pro-Kin balance test results due to the mechanism was unclear. We suppose the possible mechanisms of how the ankle stiffness affected balance function are as follows.

The central controller uses sensory information to generate descending commands that produce corrective muscle forces to stabilize the body (63). The central nervous system (CNS) injuries post-stroke leads to muscle weakness and spasticity of the affected limb(s) post-stroke, often accompanied with drop and varus foot (64, 65), and lack of mobilization and prolonged spasticity may be accompanied by structural changes of muscle fibers and connective tissue, which may result in reductions in ROM and the contact area between the sole and the ground (66). Several studies have demonstrated that higher resistance torque, increased joint stiffness, and decreased ankle ROM characterized by stroke survivors improved after completing passive stretching exercises (18–20, 22, 67, 68). One previous study reported that the weaker inter-limb coordination in the sagittal plane (DF-PF) after stroke would cause imbalance (66). The intelligent stretching decrease ankle stiffness in the sagittal plane (DF-PF), which improved inter-limb coordination post-stroke in the F/B direction. Stroke survivors usually load more over their non-paretic limb than the paretic one when standing (69), as the re-establishment of ankle strategies, balance in M/L direction was also improved (67). After stretching, loosening of stiff muscle fascicles tendon and/or aponeuroses might facilitate force generation among fascicles and increase their overall force output, which increases the passive stability of the ankle joint by limiting ankle movement in both the frontal and sagittal planes (18, 67). Alterations in muscle-tendon unit stiffness and length induced by continuous stretching could improve the fidelity of ankle proprioception (50, 60), which might improve balance. In conclusion, we assumed that the decreased DF stiffness might improve balance by activating the muscles and increasing proprioception and ROM of the ankle joints. Further studies are necessary to verify this hypothesis. Overall, in this study, a significant correlation (τ = 0.265, P = 0.041) was observed between MAS and DF stiffness, which is consistent with the previous studies (19). This measure of stiffness can be used to obtain a more quantitative evaluation of ankle properties in the future.

## Limitations

This study had limitations in methodological. First, given the number of samples available, between-group comparisons represented no differences in the biomechanical properties or balance function. Future studies with more subjects involved might show further group differences and increase the power of the study. Second, this study lacks a quantitative assessment of proprioception of the ankle joint, which is important in exploring the mechanisms of balance control recovery after stretching exercises. Third, the long-term effects of stretching training were unknown due to the absence of a follow-up period. Besides, our study only investigated the correlations between ankle stiffness and static balance in stroke survivors. The effects of ankle properties on dynamic balance need further investigation. Furthermore, muscle activations around the ankle joint will be collected using EMG for analysis as a function of ankle ROM, balance control, etc. Another limitation is the lack of a third group treated without stretching exercises. We plan to add one group of patients without stretching exercises to eliminate the improvement of ankle joint properties and balance function due to the natural history of stroke and better evaluate the effectiveness of the stretching therapy in the future. Future studies will further address these issues.

## Conclusions

The robot-aided and manual ankle stretching training provided similar significant improvements in the ankle properties, balance, motor function, and ADL post-stroke. The robot-aided stretching devices provided labor-saved, high-intensity, and well-controlled passive stretching to stroke survivors with ankle impairments, which showed additional improvements across more parameters including the spasticity and stiffness of the ankle. Findings in this study suggested that robot-aided rehabilitation may be a beneficial addition to current rehabilitation programs. As an important part of posture control, the ankle joint properties were important in keeping the upright stance. In particular, ankle stiffness was correlated with balance function post-stroke. As a biomechanical property of the ankle joint, dorsiflexion stiffness may be a sensitive indicator for evaluating the balance ability post-stroke and predicting the risk of falls in the future.

## Data Availability Statement

The datasets used and analyzed during the current study are available from the corresponding author upon appropriate request.

## Ethics Statement

The studies involving human participants were reviewed and approved by the Medical Ethics Committee of Beijing Tsinghua Changgung Hospital (18172-0-01). The patients/participants provided their written informed consent to participate in this study.

## Author Contributions

YP and L-QZ contributed to the study design, analysis, and interpretation of data and revisions to the manuscript. QW contributed to the analysis and interpretation of data. XZ contributed to the study design, data collection, analysis, interpretation of data, and drafting of the manuscript. Data collection was performed by XZ, XL, and QX. YZ and SF performed experiments. All authors read and approved the manuscript submitted and agree to be accountable for all aspects of the work.

## Funding

This study was supported by Beijing Municipal Natural Science Foundation (L182028) and Beijing Municipal Science and Technology Commission (Z181100009218003 and Z181100003118004). No additional external funding was received for this study. The funders had no role in study design, data collection, and analysis, decision to publish, or preparation of the manuscript.

## Conflict of Interest

L-QZ holds an equity position in Beijing LTK Science and Technology Co., which made the ankle rehabilitation robot used in this study. The remaining authors declare that the research was conducted in the absence of any commercial or financial relationships that could be construed as a potential conflict of interest.

## Publisher's Note

All claims expressed in this article are solely those of the authors and do not necessarily represent those of their affiliated organizations, or those of the publisher, the editors and the reviewers. Any product that may be evaluated in this article, or claim that may be made by its manufacturer, is not guaranteed or endorsed by the publisher.

## Abbreviations

ROM: Range of Motion
COP: Center of Pressure
MAS: Modified Ashworth Scale
DF: dorsiflexion
PF: plantarflexion
FM-LE: Fugl-Meyer Motor Assessment of Lower Extremity
BBS: Berg Balance Scale
MBI: Modified Barthel Index
ADL: Activities of daily living
OE: Opened eyes
CE: Closed eyes
SD: Standard deviation
AS: Average speed
M/L: Medial/Lateral
F/B: Forward/Backward
BMI: Body Mass Index
M: male
F: female
DSP: Digital signal processor
CI: Confidence interval.
